# Bovine FRAS1: mRNA Expression Profile, Genetic Variations, and Significant Correlations with Ovarian Morphological Traits, Mature Follicle, and Corpus Luteum

**DOI:** 10.3390/ani14040597

**Published:** 2024-02-12

**Authors:** Leijing Zhu, Siyuan Shen, Chuanying Pan, Xianyong Lan, Jie Li

**Affiliations:** College of Animal Science and Technology, Northwest A&F University, Yangling 712100, China; zhuleijingjingshu@163.com (L.Z.); ssy991202@163.com (S.S.); panyu1980@126.com (C.P.)

**Keywords:** bovine, *FRAS1*, variation, follicle, corpus luteum, ovary

## Abstract

**Simple Summary:**

Herein, the mRNA expression profile and genetic variations of bovine *FRAS1* detected by GWAS, as well as their association with fertility-related characteristics in unilateral ovaries from 2111 cows at the same age phase, were explored. Two deletion mutations, P3-D20-bp and P4-D15-bp, were significantly correlated with ovarian morphological traits, mature follicles, or corpora lutea in dioestrum or metaestrus. Moreover, the mRNA expression of cows with a different genotype of P3-D20-bp was also significantly different. According to binding prediction by online databases, the deletion of P3-D20-bp could disturb the binding efficiency of WT1-I and Sox2 with FRAS1 sequence, indicating that the mutation may affect gene expression levels and traits by influencing the binding of transcription factors. So, P3-D20-bp and P4-D15-bp of FRAS1 gene could be candidates for the application of MAS in optimizing female fertility in bovine breeding.

**Abstract:**

The amelioration of bovine fertility caused by a multi-factorial problem has always been a hot topic, among which the detection of available target genes is the most crucial. It was hypothesized that the Fraser extracellular matrix complex subunit 1 (*FRAS*1) gene detected by GWAS is involved in physiological activities such as ovarian development. Herein, unilateral ovaries from 2111 cows were used to examine the mRNA expression profile and polymorphisms of bovine *FRAS1* and their associations with fertility-related characteristics. Firstly, it was confirmed that *FRAS1* gene transcripts are expressed in various bovine tissues. Then, among five potential insertion–deletion (indel) loci, the 20 bp (named P3-D20-bp) and 15 bp (P4-D15-bp) deletion mutations were confirmed to be polymorphic with linkage equilibrium. Secondly, the P3-D20-bp polymorphism was significantly associated with ovarian weight and corpus luteum diameter in the metaestrus phase and ovarian length in the dioestrum stage. Additionally, both ovarian length and mature follicle diameter in metaestrus are significantly correlated with different genotypes of P4-D15-bp. Thirdly, the transcriptional expression of the *FRAS1* gene in groups with a minimum value of ovarian weight or volume was significantly higher than the expression in groups with a maximum value. Instead of that, the more corpus luteum and mature follicles there are, the higher the transcription expression of the *FRAS1* gene is. Furthermore, *FRAS1* expression in cows with a heterozygous genotype (ID) of P3-D20-bp was significantly higher than others. Eventually, P3-D20-bp deletion could disturb the binding efficiency of WT1-I and Sox2 to *FRAS1* sequence according to binding prediction, indicating that mutation may affect gene expression and traits by influencing the binding of transcription factors. Overall, the polymorphisms of P3-D20-bp and P4-D15-bp of the bovine *FRAS1* gene significantly correlated to follicle or ovarian traits that could be applied in optimizing female fertility in cow MAS breeding programs.

## 1. Introduction

Globally, certain breeding goals focused on enhancing milk yield subsequently result in a negative impact on cattle fertility or diseases susceptibility [1,2]. As a complex feature involving multiple events, female reproduction is influenced by multiple genetic factors [3]. Identifying the biological processes involved in the regulation of complex traits is challenging because they are regulated by multiple genes, each of which contributes little to overall genetic variance [4]. Recently, numerous studies have revealed potential molecular markers and underlying genetic mechanisms of bovine complex traits through genome-wide association studies (GWAS), which include fertility-related traits [5,6,7,8]. Importantly, several candidate genes for different quantitative trait loci (QTL) have been identified based on GWAS [9], confirming that the Fraser syndrome protein 1 (*FRAS1*) may be a candidate gene in terms of pregnancy and bovine fertility [4].

*FRAS1*, as an extracellular matrix protein, adheres between the epidermal basement membrane and the underlying dermal connective tissue during embryonic development [10]. *FRAS1* mutations can lead to an autosomal recessive malformation syndrome known as Fraser syndrome (FS), characterized by occlusion, syndactyly, and reproductive system defects [11,12,13]. FRAS1 binds to *FREM1*, *FREM2,* or other matrix molecules to provide early adhesion during basement membrane formation [13,14,15], and loss of its function leads to extracellular matrix (ECM) defects in the second trimester and impaired transforming growth factor β (TGF β) family signaling, or others, and most of the signaling molecules of TGF-β superfamily are involved in follicular development [15,16]. In addition to the important functions of *FRAS1* in embryonic development, pregnancy, and fetal growth described above, *FRAS1* also affects the treatment of ovarian cancer as an essential genomic locus [17], and there is a significant correlation between *FRAS1* mutations and antenatal/perinatal mortality [18]. So, *FRAS1* has important biological functions in the reproductive process and embryonic development process, but it is unknown whether *FRAS1* will influence bovine ovarian development and bovine fertility.

The establishment and maintenance of pregnancy involves a complex interaction among the endometrium, embryo, and corpus luteum (CL) [19]. An intact and highly efficient reproductive tract is a key element for the success of a bovine breed. For the female, the ovary is the vital source for oocyte release, and the remaining somatic cells in the follicle will undergo terminal differentiation to form the corpus luteum (CL) after oocyte release [20,21], which primarily secretes progesterone and is essential for the establishment and maintenance of pregnancy. In the absence of pregnancy, various indicators, such as CL degeneration to corpus albicans [22], ovarian development, and mature follicle quality, can be used to evaluate the reproductive performance of cows [23,24,25].

Therefore, it was hypothesized that *FRAS1*, the candidate gene identified by GWAS associated with cow fertility, is closely correlated to ovarian development or related progress, so several important phenotypes, such as ovarian phenotype, the phenotype of corpus luteum, and follicle, in healthy Holstein were selected for correlation analysis, and quantitative analysis was performed in tissues with different traits to explore the mRNA expression profile of *FRAS1* and find out whether *FRAS1* has an important influence on bovine fertility, which can be a novel molecular marker of general trait such as fertility or ovarian development for marker-assisted selection in bovine genetic breeding.

## 2. Materials and Methods

### 2.1. Collection of Bovine Ovary Tissues

Herein, a total of 2111 unilateral ovaries were sampled from different healthy adult Holstein cows at the same age phase with the same feeding and management conditions. Our previous study sequenced the complete mitochondrial DNA D-loop region of 501 ovaries and found two haplogroups in this tested population, which also identified all the collected ovaries from different cows [26]. Ovarian morphological phenotypes (ovarian length and weight, etc.), follicle, or corpus-related traits were measured using a standard protocol reported by [27]. Among these, corpora lutea were further classified according to their shape: type I luteum is conical, type II is crater-shaped, type III is mushroom-shaped, and type IV is flat [28].

According to the type of luteum and the presence or absence of follicles, the estrous cycle phases of the sampled cows were divided into four groups: cows with luteal type I or II were judged to be in metaestrus; cows with type III or IV CL were in dioestrum; females with no follicles or luteum on their ovaries or only corpus albicans (luteal degeneration and atrophy) were judged to be in proestrus; and cows with no luteum and large follicles on their ovaries were considered to be in estrus. All the experimental procedures used in this study followed the principle of the International Animal Care and Use Committee of Northwest A&F University (protocol number: NWAFAC1008).

### 2.2. Potential Indel Loci Screening, Identification, and Genotyping

Bovine genomic DNA samples were extracted from ovarian cortex composed of follicles and connective tissue by a mature high-salt method [29], and all DNA samples were diluted to 10 ng/µL as a working concentration after testing their concentration and purity on a NanoDrop 1000 (Thermo Scientific, Waltham, MA, USA).

Given the convenience and timeliness of detection, we specifically focused on potential indel mutation of the bovine *FRAS1* gene. Five potential insertion/deletion (indel) loci within the bovine *FRAS1* gene were selected based on the Ensembl database (http://asia.ensembl.org/index.html, accessed on 11 February 2024). The corresponding amplification primers were designed by the NCBI online tool based on the bovine *FRAS1* gene sequence (Gene ID: 107131184, NC_037333.1 92954024…93295487, Table 1) and were randomly synthesized by Sangon Biotech (Shanghai, China). Thirty samples of DNA were randomly selected and mixed for PCR testing to detect the presence of polymorphism at five selected loci and to further estimate their mutation frequencies. We followed the PCR amplification reaction system and procedure (touch-down) outlined in our previous reports, and PCR products were separated on 3.5% agarose gels with TBE buffer [30], and then the products of each primer showing different genotypes were sequenced.

After performing the above steps, two indel (rs522341234 and rs453892138) were identified to be polymorphic among the five potential indel loci. Their related mutation frequencies were preliminarily estimated to be greater than 1%, which is worth further investigation in the population, so, in this study, two polymorphic loci were amplified and genotyped in 2111 cows.

### 2.3. Extraction of Total RNA and cDNA for qRT-PCR

The total RNA was extracted from various tissues of adult cows by the RNAiso plus-trichloromethane method. Within each tissue type, more than 3 different cows were collected, and the corresponding cDNA was reversed by a reverse transcription kit (TaKaRa, Beijing, China). Sequences of the bovine *FRAS1* and β-actin for quantitative real-time PCR (qRT-PCR) (listed Table 1) were designed by the PrimerQuest Tool (https://sg.idtdna.com/Primerquest/Home/Index, accessed on 11 February 2024). As for qRT-PCR analyzing the expression of *FRAS1*, each treatment group contained 3 biological replicates (different cows) with 3 technical replicates of each cow.

### 2.4. Detection of Whether Expression Levels Are Associated with Mutations

According to the values of ovarian weight, ovarian volume, the number of mature follicles, and CL, certain ovaries were selected and divided into four groups based on either the maximum or minimum values, respectively. The differences between the maximum and minimum groups are highly significant, and specific P-values can be referenced from previous work in 2021 [27]. Moreover, nearly 50 different ovaries were selected for RNA extraction and further used for qRT-PCR analysis based on the maximum and minimum values of the above four traits. To explore whether the detected mutations affect the transcription expression of the *FRAS1* gene, the mRNA expressions of *FRAS1* of cows with different genotypes were explored. The reaction mixtures and qRT-PCR conditions were the same as previously described [30].

### 2.5. Statistical Analysis and Transcription Factor Binding Prediction

The chi-square (χ^2^) test was used to calculate the genotypic and allelic frequencies of two indel mutations within the bovine *FRAS1*, and the values of polymorphism information content and Hardy–Weinberg equilibrium were calculated by Nei’s method or the GDIcall Online tool (http://www.msrcall.com/Gdicall.aspx, accessed on 11 February 2024). Additionally, the SHEsis program (http://analysis.bio-x.cn, accessed on 11 February 2024) was used for the linkage disequilibrium and to calculate haplotypes [31].

In this study, an adjusted linear model with fixed effects, Y_ij_ = μ + G_i_ + E_ij_, where Y_ij_ is the trait measured on each of the ijth animal; μ is the overall mean; G_i_ is the type of ith genotype; and E_ij_ is the random error term, was used to assess the relationships between genotypes and morphological traits or others. Farm, season of birth, age, sex, and breed are not variable or influencing factors in this study, so they were not used in the reduced linear model. Furthermore, an independent-samples *t*-test and analysis of variance (ANOVA), implemented in SPSS software (v24.0; IBM Corp., Armonk, NY, USA), were used to analyze the association of the genotypes with all ovarian traits of interest. Moreover, the combinations of transcription factors (TFs) with sequences in the mutant regions were predicted by Alibaba2 (gene-regulation.com/pub/programs/alibaba2/index.html, accessed on 11 February 2024) and ALGGEN (http://alggen.lsi.upc.es/, accessed on 11 February 2024) online software.

## 3. Results

### 3.1. The Transcription Expression of the FRAS1 Gene in Bovine Different Tissues

**Figure 1:**
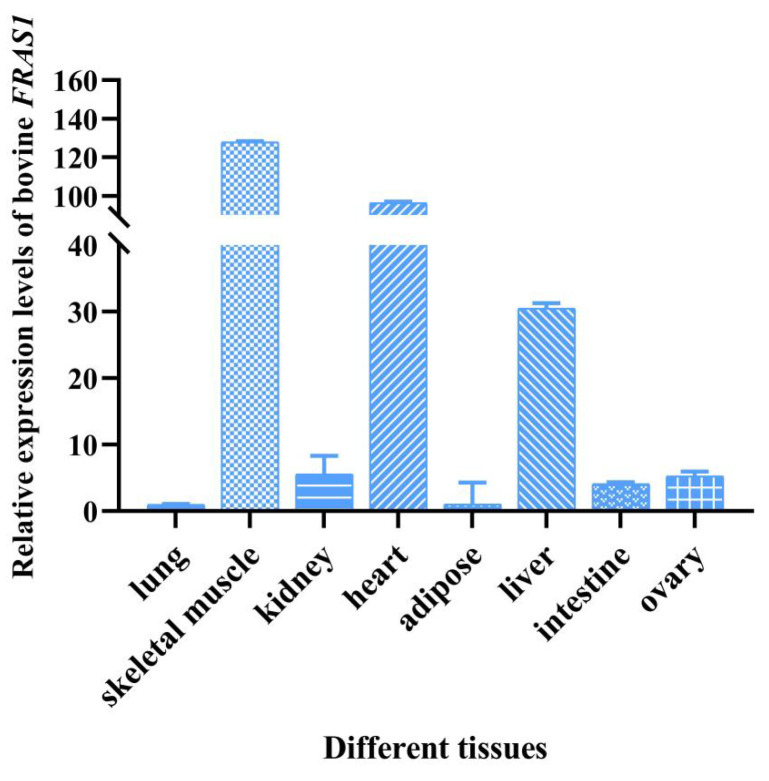
Relative expression levels of the bovine *FRAS1* mRNA in various tissues.

According to the Genebank of the NCBI database, *FRAS1* gene is expressed in a broad spectrum in both human and mouse. To further detect the transcription level of *FRAS1* gene in various bovine tissues, quantitative experiments were used, and the results showed that the mRNA expression of *FRAS1* was highest in bovine skeletal muscle, followed by heart, liver, kidney, ovary, small intestine, and adipose tissue, and lowest in cow lung (Figure 1). For these tested tissues in human, the transcription level of *FRAS1* decreased successively in human kidney, lung, heart, small intestine, fat, liver, and ovary (https://www.ncbi.nlm.nih.gov/gene/80144, accessed on 11 February 2024), while the mRNA expression level of *FRAS1* gene decreased successively in mouse kidney, ovary, lung, liver, heart, and small intestine (https://www.ncbi.nlm.nih.gov/gene/231470, accessed on 11 February 2024). Therefore, *FRAS1* gene is expressed in ovarian tissues of various species, such as mouse, human, and cow.

### 3.2. Identification of the P3-D20-bp (rs522341234) and P4-D15-bp (rs453892138) Polymorphisms

**Figure 2:**
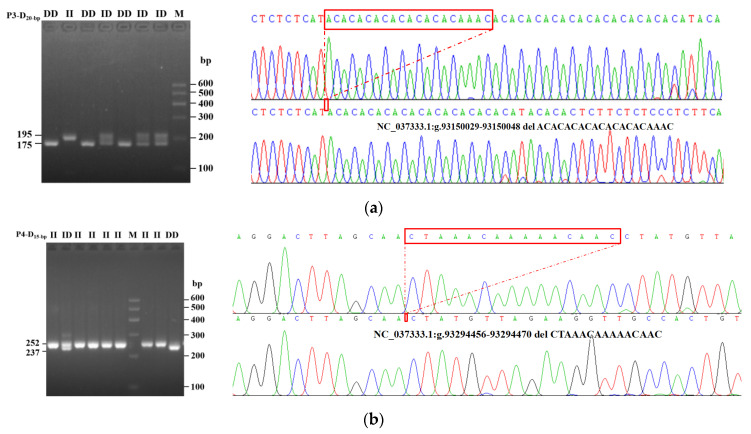
The graphics of the (**a**) P3-D20-bp (rs522341234) and (**b**) P4-D15-bp (rs453892138) loci identified by agarose gel and DNA direct sequencing.

Considering the operability and efficiency of testing, five potential sites with mutated fragments greater than 10 bp were selected and identified (Table 1). Among them, both 20 bp deletion (rs522341234) and 15 bp deletion (rs453892138) were confirmed to be polymorphic and were named as P3-D20-bp and P4-D15-bp, respectively (Figure 2). Moreover, P3-D20-bp, NC_037333.1: g.93150029-93150048 del ACACACACACACACACAAAC is located in intron 29 with three genotypes in the test population, insertion/insertion (II, 195 bp), insertion/deletion (ID, 195/175 bp), and deletion/deletion (DD, 175 bp) genotype (Figure 2a), while P4-D15-bp, NC_037333.1:g.93294456-93294470 del CTAAACAAAAACAAC is located in the non-coding region of exon 73 in bovine *FRAS1* gene with three genotypes; similarly, there is also II (252 bp) genotype, ID (252/237 bp) genotype, and DD (237 bp) genotype (Figure 2b).

### 3.3. Genetic Parameters and Haplotype Analysis of Two Polymorphic Indels

After the detection and locus genotyping of cows, population polymorphism indicators of P3-D20-bp and P4-D15-bp were calculated, including allele frequency, polymorphism information content (PIC), etc. As shown in Table 2, P3-D20-bp has a higher mutation frequency in the tested population because the value of the mutant allele “D” frequency was 0.729, while the frequency value of the wild genotype II was only 0.079. The effective allele number (Ne) of P3-D20-bp was 1.652, which is close to the absolute value for the alleles (2), indicating that its alleles are more evenly distributed in the population than P4-D15-bp, for which the Ne value was only 1.350. As for P4-D15-bp, the value of the mutant allele “D” frequency was 0.153, and the frequency value of the wild genotype II was 0.774. Moreover, the polymorphic information content values of P3-D20-bp and P4-D15-bp were 0.317 and 0.226, respectively.

**Figure 3:**
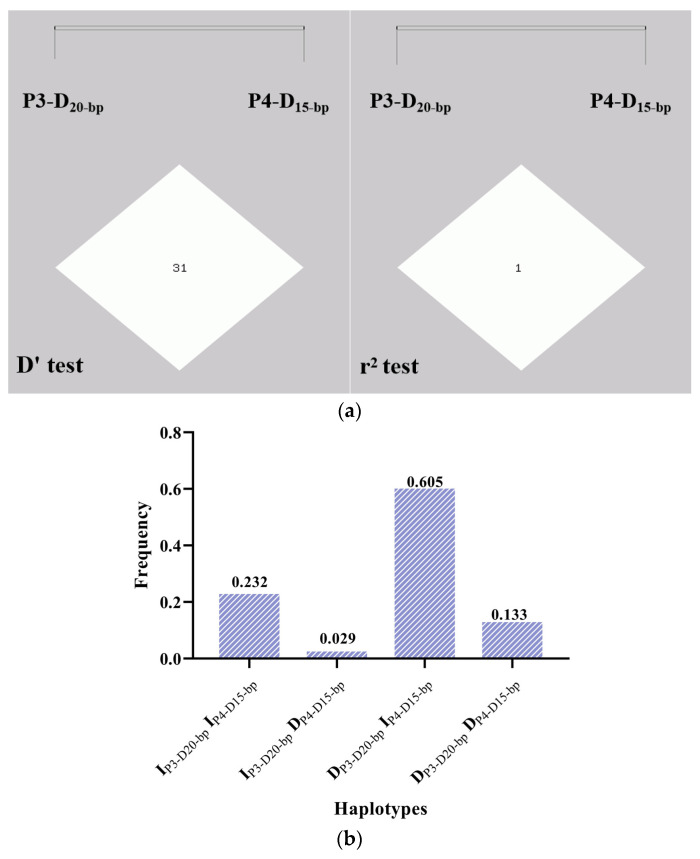
(**a**) Analysis of linkage disequilibrium and (**b**) haplotype frequencies of the P3-D20-bp (rs522341234) and P4-D15-bp (rs453892138) alleles within the *FRAS1* gene. Note: D and I mean deletion and insertion allele, respectively.

In addition, both the D’ test and r^2^ test showed that there was no linkage imbalance between the two loci, and with values of 0.310 and 0.007, respectively, revealing that the two pairs of alleles follow the law of independent association (Figure 3a). In addition, the two pairs of alleles could freely form four types of haplotypes, and DP3-D20-bp IP4-D15-bp have the highest frequency with 0.605, followed by IP3-D20-bp IP4-D15-bp with a frequency value of 0.232, DP3-D20-bp DP4-D15-bp with 0.133, and the lowest frequency of IP3-D20-bp DP4-D15-bp genotype was 0.029 (Figure 3b).

### 3.4. Correlation Analysis of Two Indel Polymorphisms with Ovarian Dimensions Phenotypes

To further verify whether the *FRAS1* gene can be used as a candidate molecule for cow fertility or ovarian development screening [4], the association between the two tested indels of *FRAS1* and the indicators related to ovarian dimension was analyzed.

In the dioestrum group, P3-D20-bp polymorphisms are significantly associated with ovarian length (*p* = 0.008), and the ID genotype was the most dominant genotype, while cows with genotype II had the smallest length value (Table 3). However, this significant association did not exist in the other stage groups (Table 3).

Coincidentally, ovarian length was also significantly associated with P4-D15-bp polymorphism in the metaestrus group (*p* = 0.045), and the most dominant genotype was ID, which is consistent with P3-D20-bp at dioestrum (Table 4). Except for the metaestrus group, there was no significant relationship between P4-D15-bp polymorphism and phenotypic traits of the ovary (Table 4).

### 3.5. Relationships between the Tested Polymorphisms of FRAS1 and Mature Follicles, Luteum, or Corpus Albicans

Compared with ovarian dimension traits, an indirect indicator of fertility evaluation, follicular and luteal traits can more directly reflect female fertility, so we further focused on revealing the relationship between polymorphisms of *FRAS1* and the above traits. In the metaestrus stage, different polymorphisms of P4-D15-bp were significantly correlated with mature follicle diameter (*p* = 0.004), and the dominant genotype was DD (Table 4).

In addition, different polymorphisms of P3-D20-bp have statistical significance with the CL diameter (*p* = 0.035), and wild genotype II cattle were associated with the smallest diameter of CL than the others (Table 3). No significant relationship was found between the examined two polymorphisms and follicular number, number of luteum, or corpus albicans in each group (Table 3 and Table 4).

### 3.6. Correlations between Ovarian Traits and mRNA Expression of the FRAS1 Gene

**Figure 4:**
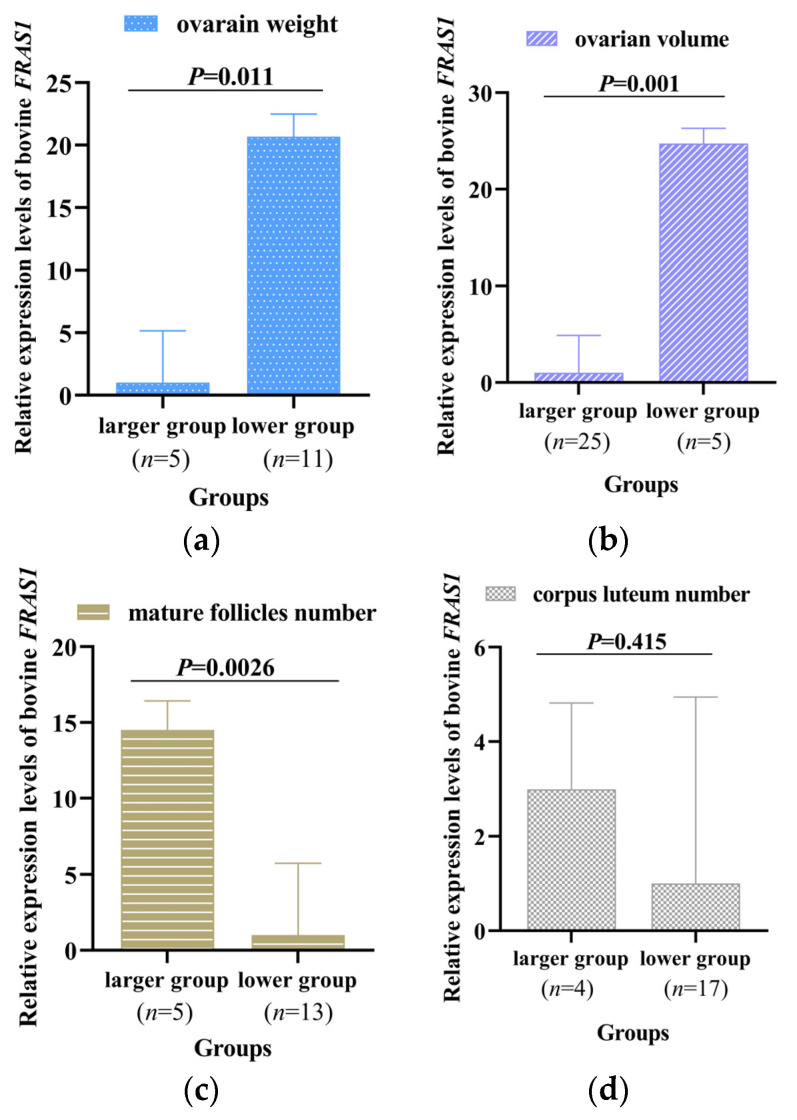
The mRNA expression difference between cows with the maximum and the minimum groups of (**a**) ovarian weight, (**b**) volume, (**c**) the number of mature follicles, and (**d**) corpus luteum.

To further explore the reasons for the significant correlation of *FRAS1* gene polymorphisms with follicular, luteal, and ovarian related traits, the transcription expression levels of the *FRAS1* gene were investigated in the maximum and minimum groups of ovarian weight and volume, the number of mature follicles, and CL. The mRNA level of the *FRAS1* gene in the lower groups of ovarian weight (*p* = 0.011, Figure 4a) and volume (*p* = 0.001, Figure 4b) was significantly higher than in the higher groups. In contrast, the more mature the follicles and CL, the lower the transcription level of the *FRAS1* gene, and the expression level of the highest group was higher than that of the lowest group of mature follicles (*p* = 0.0026, Figure 4c) and CL (*p* = 0.415, Figure 4d).

**Figure 5:**
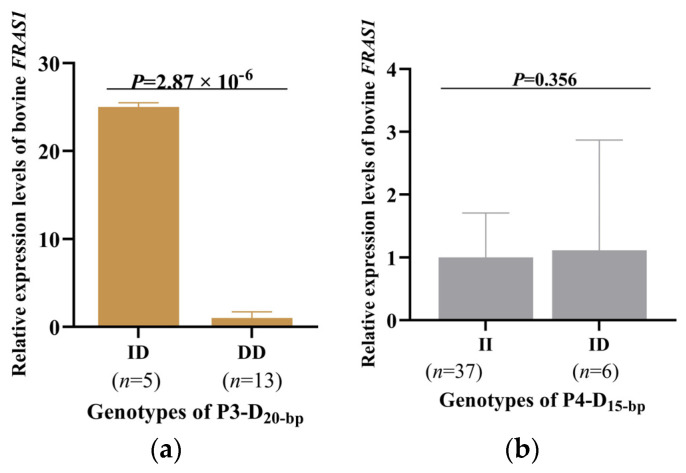
Relative expression of the bovine *FRAS1* mRNA in the different genotype groups of (**a**) P3-D20-bp (rs522341234) and (**b**) P4-D15-bp (rs453892138).

Furthermore, to determine whether mutations affect gene transcription, the mRNA levels of *FRAS1* were further explored in different groups within different genotypes. For P3-D20-bp, the mRNA of cows with the ID genotype was significantly higher than others with DD (*p* = 2.87 × 10^−6^, Figure 5a). Conversely, as for P4-D15-bp, the expression difference between heterozygous mutants (ID) and wild-type cows was not significant (Figure 5b).

### 3.7. Potential Combination of Transcription Factors with a Mutation Sequence

**Figure 6:**
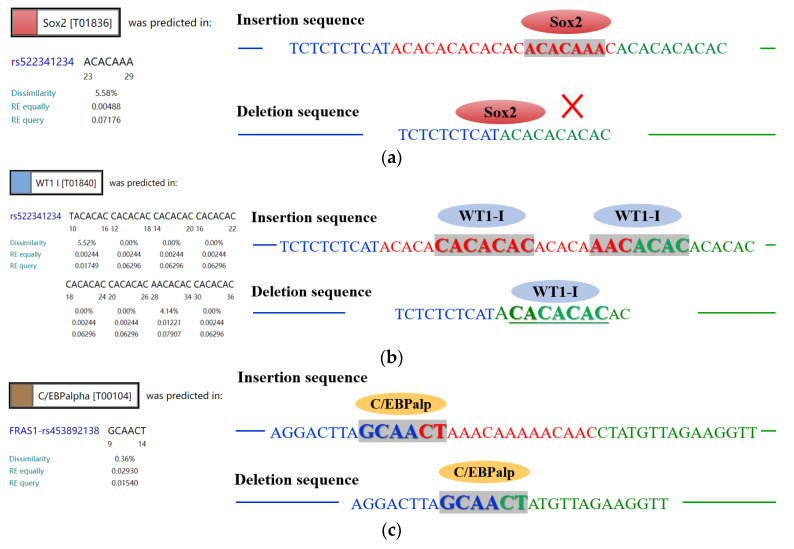
Combine forecast of transcription factors (TFs) with mutation sequence of (**a**,**b**) P3-D20-bp (rs522341234) and (**c**) P4-D15-bp (rs453892138) of the *FRAS1* gene.

To confirm whether the detected mutations disrupt the binding of the transcription factors with mRNA, thereby affecting *FRAS1* gene expression, the online databases of ALGGEN and Alibaba2 were used to predict binding. Herein, the P3-D20-bp deletion mutation could affect the binding efficiency of WT1-I (T01840) and disturb the binding of Sox2 (T01836) to *FRAS1* sequence (Figure 6a,b). However, for the P4-D15-bp mutation located in the non-coding region of exon 73, only one transcription factor, C/EBPα (T00104), was predicted to bind at this site, but binding efficiency was not affected by the 15 bp deletion mutation (Figure 6c).

## 4. Discussion

Female fertility, with a great impact on production, has received much attention because it is directly related to the ability to produce offspring necessary to offset costs in production systems in cattle production [3]. As the female’s vital endocrine organ producing estrogen, progesterone, and a small amount of androgens, the ovary performs multiple physiological functions, including ovulation, follicular formation, oocyte loss/selection, and atresia, which are important for optimal fertility. Gonad development is closely related to female reproductive capacity, among which the dimension and weight of ovary play a crucial role in reproduction. As reported, histology studies confirm that the follicles number and dimension, and the occurrence of atretic follicles, were related to ovarian weight and size of ovary in wild boar, so the macroscopic evaluation of ovaries is a valid method for the assessment of reproductive status [32]. Considering the importance of ovarian dimensions and follicular number on fertility, several candidate genes, including septin 7 [33], DENN domain containing 1A [34], integrin β5 [35], hydroxysteroid 17-beta dehydrogenase 3 (HSD17B3), and adenylate cyclase 5 (ADCY5) [30], which are closely related to fertility and regulate ovarian phenotypic traits, have been identified and verified based on candidate gene strategy.

According to a previous GWAS report [4], *FRAS1* was selected as a candidate gene for bovine fertility, and its polymorphisms have been proven to have a significant correlation with ovarian, follicles, or CL traits, consistent with previous verified ITGB5 and ADCY5. Fras1 has become known for its mutations, resulting in sub-epidermal blistering and the fusion of eyelids and digits, as well as the malformation of lungs and kidneys, also known as Fraser syndrome in humans [36]. However, in addition to the above phenotype, mutations in the pig counterpart *FRAS1* are responsible for potentially affecting feed efficiency [37] and growth traits in pigs [38]. Herein, polymorphisms of the bovine *FRAS1* gene were proven to be significantly associated with follicle or ovarian traits. As an extracellular-matrix-associated protein, Fras1 plays an important role in epithelial and mesenchymal adhesion during early embryonic development [39,40]. A polymorphism in the promoter of *FRAS1* is a candidate SNP associated with metastatic prostate cancer [41], and *FRAS1* mRNA was highly expressed in tissues rich in muscle cells or fibers in this study. A mutation in the *FRAS1* gene serves as the fundamental trigger for Fraser syndrome, leading to prenatal mortality in human fetuses [42]. However, there is scarce research on the FRAS1 gene in livestock animals currently. Nevertheless, based on GWAS, FRAS1 is considered not only a reproduction-related gene in cattle but also in pigs [4,43], and this perspective is further supported by the findings of the present study. Thus, it could affect the development of ovarian morphology by influencing the formation of smooth muscle fibers extended with the ovarian ligament. Additionally, given that the *FRAS1* gene encodes an extracellular matrix protein, it can be inferred that the detected mutation may affect cell migration or other processes, thereby influencing the formation and release of mature follicles as well as CL formation.

Furthermore, the expression trend of the *FRAS1* gene shows a negative correlation with ovarian weight (Figure 4). Moreover, deletion mutations in *FRAS1* gene segments lead to a reduction in the binding affinity of TFs, thereby inhibiting FRAS1 gene transcription (Figure 6). The *FRAS1* expression level for the DD genotype was significantly higher than that for the ID genotype in P3-D20-bp, suggesting that this may be attributed to a higher TFs binding inhibition rate in homozygous deletion compared to heterozygous deletion. This inference was consistent with the trends observed in Table 3, where individuals with the DD genotype, exhibiting the highest TFs inhibition rate, also have the maximum ovarian weight. As a limit of this study, the above speculations have not been confirmed by the experiments in this study, so the further progression of relevant studies is still needed.

In recent decades, genetic improvements in quantitative traits have been achieved by selecting genetically superior parents using phenotypic and pedigree information through BLUPs. However, in recent years, increasingly sophisticated MAS breeding, genomic selection, and genetic modification have rapidly replaced traditional breeding techniques due to the rapid development of international livestock and poultry industrialization. For complex economic traits such as fertility, thousands of candidate genes and molecular markers yet to be identified have been mined by various omics techniques, and already some of them have been identified and used in livestock breeding, such as solute carrier organic anion transporter 1B3 (SLCO1B3) and growth hormone receptor (GHR) genes in chicken breeding [44]. In dairy cattle breeding programs, MAS not only incorporates molecular information on detected QTLs in BLUP breeding schemes but also increases genetic merit due to an increased accuracy of genetic evaluation, especially in situations where selection based on best linear unbiased prediction (BLUP) evaluations has limitations [45]. As previously reported, the use of markers such as SIRT1, SIRT2, LPL, CRTC2, SIX4, UCP, and ZBTB38 as selection criteria for cattle body measurements and meat quality traits is considered appropriate for use in beef cattle breeding programs [46]. Additionally, given that MAS procedures using only a few DNA markers to track a limited number of QTLs may be of low benefit, the current concept of genomic selection as an alternative technique using available dense single nucleotide polymorphism (SNP) information has been proposed in the MAS process [47]. Furthermore, in future practical cattle breeding processes with longer breeding cycles, the implementation of MAS will be closely integrated with gene editing techniques (such as CRISPR/Cas9) [48], artificial insemination, and embryo-transfer-assisted reproductive technologies [49], and the institute of the screening and verification of molecular markers is the premise for the above technical implementation; therefore, it is of great economic value in bovine husbandry.

## 5. Conclusions

Overall, two highly polymorphic indel mutations of the bovine *FRAS1* gene were significantly associated with ovarian phenotype or follicle traits, which could be a potential molecular marker for the practical selection of high-fertility cows for MAS in cow breed.

## Figures and Tables

**Table 1:** Primer sequences for the bovine *FRAS1* mutation loci amplification and qRT-PCR.

Loci	Rs Numbers	Primer Sequences (5′–3′)	Product Sizes (bp)	*Tm* (°C)	Region	Note
P1-D_36-bp_	rs438887427	F1: TTCCACTGTTTCCCCATCTATT	291/255	58.1	intron 14	deletion
		R1: GGCTGTATTTTGTCACCCTTCT				
P2-D_21-bp_	rs432223525	F2: GCATCCCTGGAATAAACCCAAT	175/154	62.0	intron 19	deletion
		R2: ACCACTACCCTGATACCAAAAC				
P3-D_20-bp_	rs522341234	F3: GTTAATCGCCCAATATGTCTCGTG	195/175	62.9	intron29	deletion
		R3: CTGAAAGAAGCCTCTCTACCACTC				
P4-D_15-bp_	rs453892138	F4: ACAGAATTCTCTCCAGAGCAATGAA	252/237	60.3	exon73 (non-coding region)	deletion
		R4: CTGTCTTGGAAGAAACAGTGGC				
P5-D_18-bp_	rs527003260	F5: GGTCGCAAAGAATTGGACACG	248/230	60.4	exon 73	deletion
		R5: TTGGCAGGTGGGTTCTTAACT				
Bovine-*FRAS1*	-	F: CACCAGGAGCTGGAATTCAT	105	62.0	-	qRT-PCR
		R: AGTCCTCCCATCTTGAAACAC				

**Table 2:** Population genetic parameters of the P3-D20-bp and P4-D15-bp polymorphisms in the bovine *FRAS1* gene.

Loci	Sizes	Genotypic Frequencies			Allelic Frequencies		HWE *p* Values	Population Parameters			
		DD	ID	II	D	I		Ho	He	Ne	PIC
P3-D_20-bp_	1169	0.537	0.384	0.079	0.729	0.271	0.350	0.605	0.395	1.652	0.317
P4-D_15-bp_	1826	0.080	0.146	0.774	0.153	0.847	1.747 × 10^−77^	0.741	0.259	1.350	0.226

Note: HWE, Hardy–Weinberg equilibrium; Ho, homozygosity; He, heterozygosity; Ne, effective allele numbers; PIC, polymorphism information content.

**Table 3:** Relationship between cow ovarian-related traits and different genotypes of P3-D20-bp.

Stage	Sizes *	Traits	Observed Genotypes (LSM ± SE)			*p* Values
			II	ID	DD	
Dioestrum	236	Ovarian weight (g)	10.21 ^b^ ± 0.55 (21)	11.93 ^b^ ± 0.49 (91)	13.64 ^a^ ± 0.39 (124)	0.019
	231	Corpus luteum diameter (mm)	14.40 ^b^ ± 1.42 (21)	17.24 ^b^ ± 0.67 (89)	18.91 ^a^ ± 0.81 (121)	0.035
	645	Ovarian length (mm)	39.33 ^B^ ± 1.03 (46)	43.20 ^A^ ± 0.52 (256)	41.87 ^AB^ ± 0.46 (343)	0.008

Note: * refers to the number of individual cattle analyzed. The capital letters (e.g., A,B) and lowercase letters (e.g., a,b) represent great significant difference (*p* < 0.01) and significant difference (*p* < 0.05), respectively.

**Table 4:** Relationship between cow ovarian-related traits and different genotypes of P4-D15-bp.

Stage	Sizes *	Traits	Observed Genotypes (LSM ± SE)			*p* Values
			II	ID	DD	
Metaestrus	322	Ovarian length (mm)	41.69 ^b^ ± 0.59 (251)	44.85 ^a^ ± 1.01 (48)	44.26 ^ab^ ± 1.52 (23)	0.045
	110	Mature follicle diameter (mm)	12.11 ^B^ ± 0.45 (85)	11.95 ^B^ ± 0.85 (14)	16.59 ^A^ ± 1.44 (11)	0.004

Note: * refers to the number of individual cattle analyzed. The capital letters (e.g., A,B) and lowercase letters (e.g., a,b) represent great significant difference (*p* < 0.01) and significant difference (*p* < 0.05), respectively.

## Data Availability

The data presented in this study are available in this article.
